# Supplementary material for: Transcriptome Profiling Provides Insights into the Early Development of Tiller Buds in High- and Low-Tillering Orchardgrass Genotypes
Source: Int J Mol Sci. 2023 Nov 15;24(22):16370. doi: 10.3390/ijms242216370 (PMC10671593; doi:10.3390/ijms242216370)
Supplement: Supplementary file 1 [file ijms-24-16370-s001.zip › Supplementary Figures.pdf]

Supplementary Figures

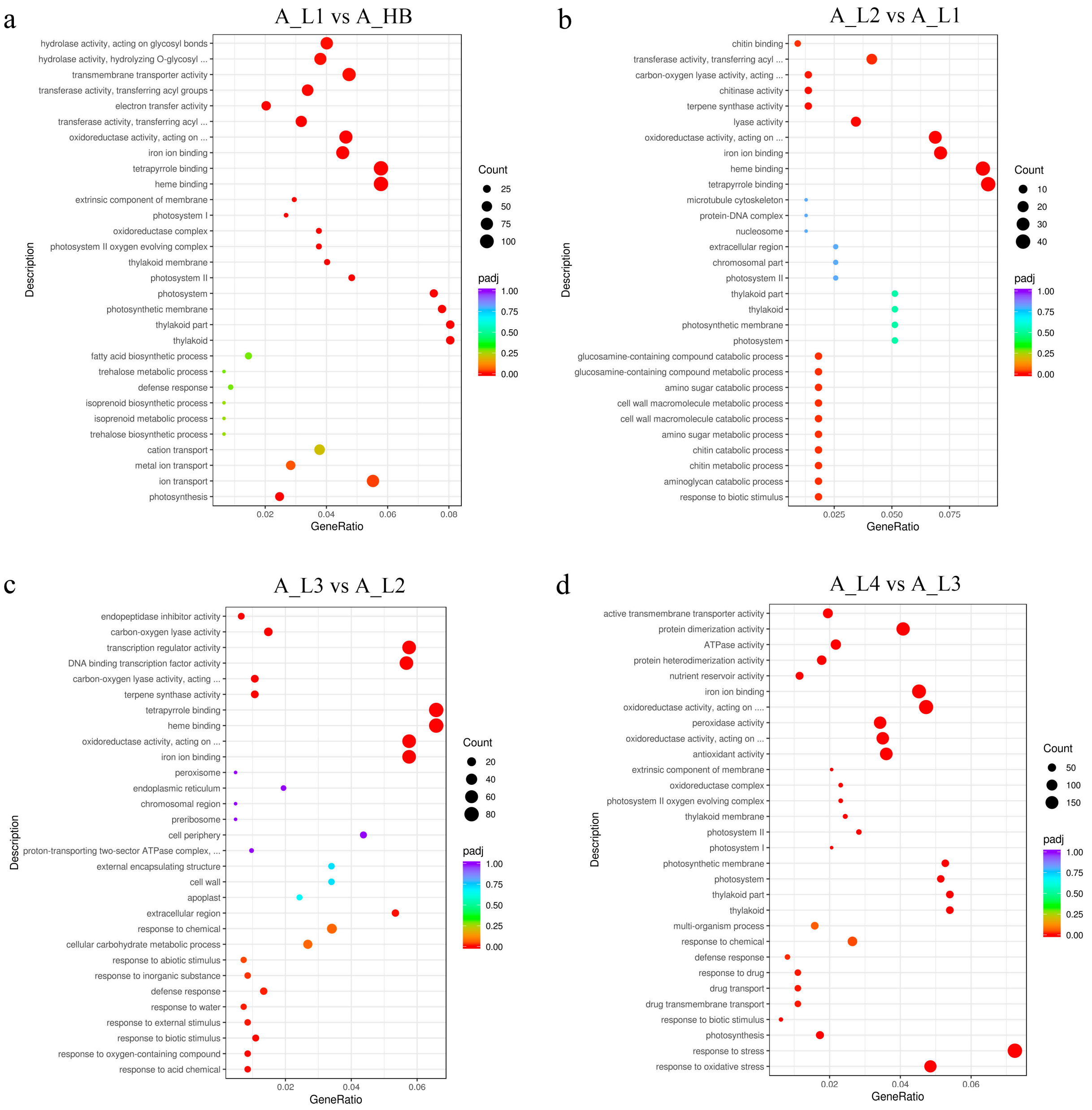

Figure S1. Gene ontology (GO) enrichment analysis of the high-tillering genotypes (AKZ-NRGR667). (a) GO enrichment of DEGs between A_L1 vs A_HB; (b) GO enrichment of DEGs between A_L2 vs A_L1; (c) GO enrichment of DEGs between A_L3 vs A_L2; (d) GO enrichment of DEGs between A_L4 vs A_L3.

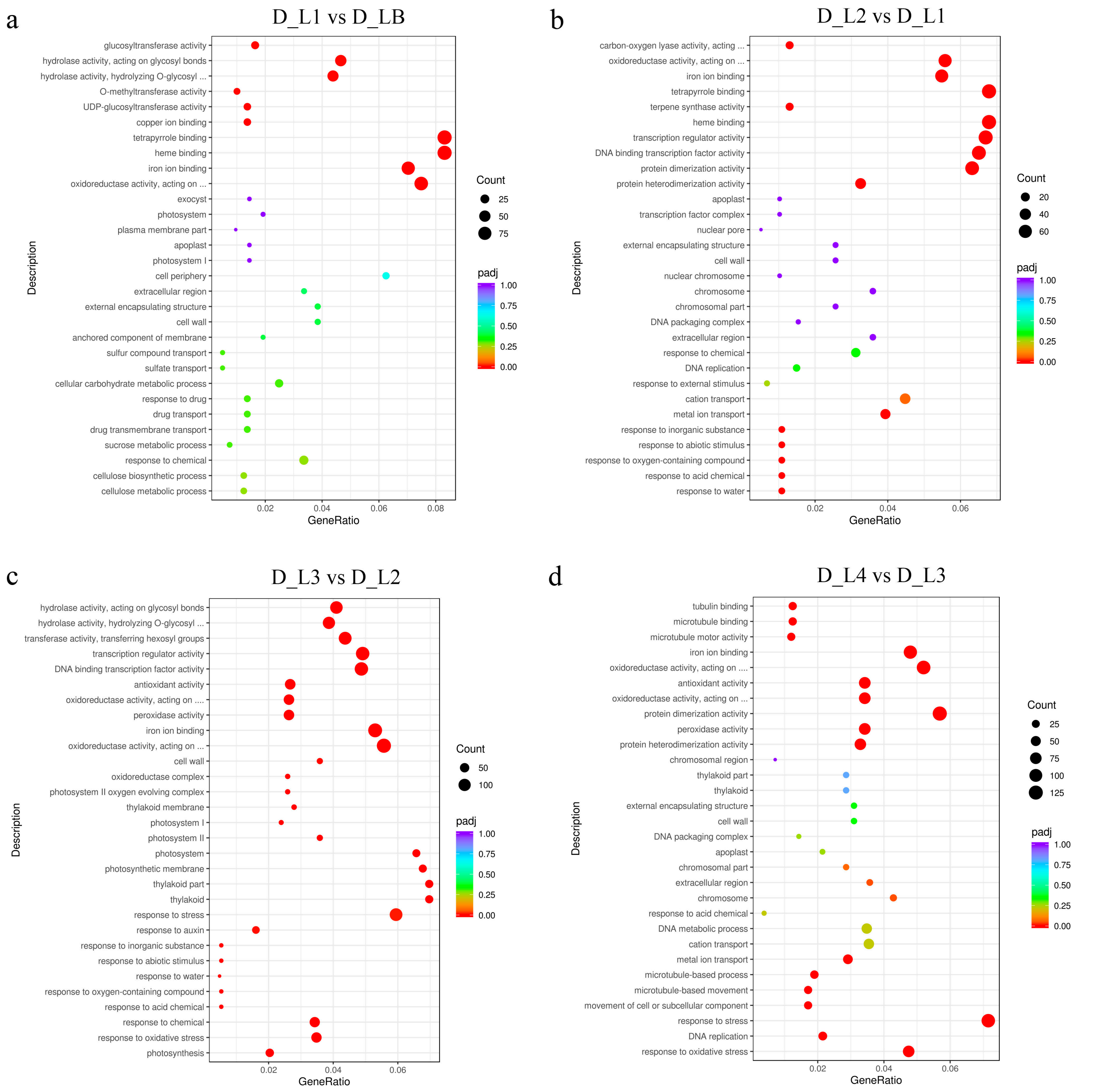

Figure S2. Gene ontology (GO) enrichment analysis of the low-tillering genotypes (D20170203). (a) GO enrichment of DEGs between D_L1 vs D_HB; (b) GO enrichment of DEGs between D_L2 vs D_L1; (c) GO enrichment of DEGs between D_L3 vs D_L2; (d) GO enrichment of DEGs between D_L4 vs D_L3.

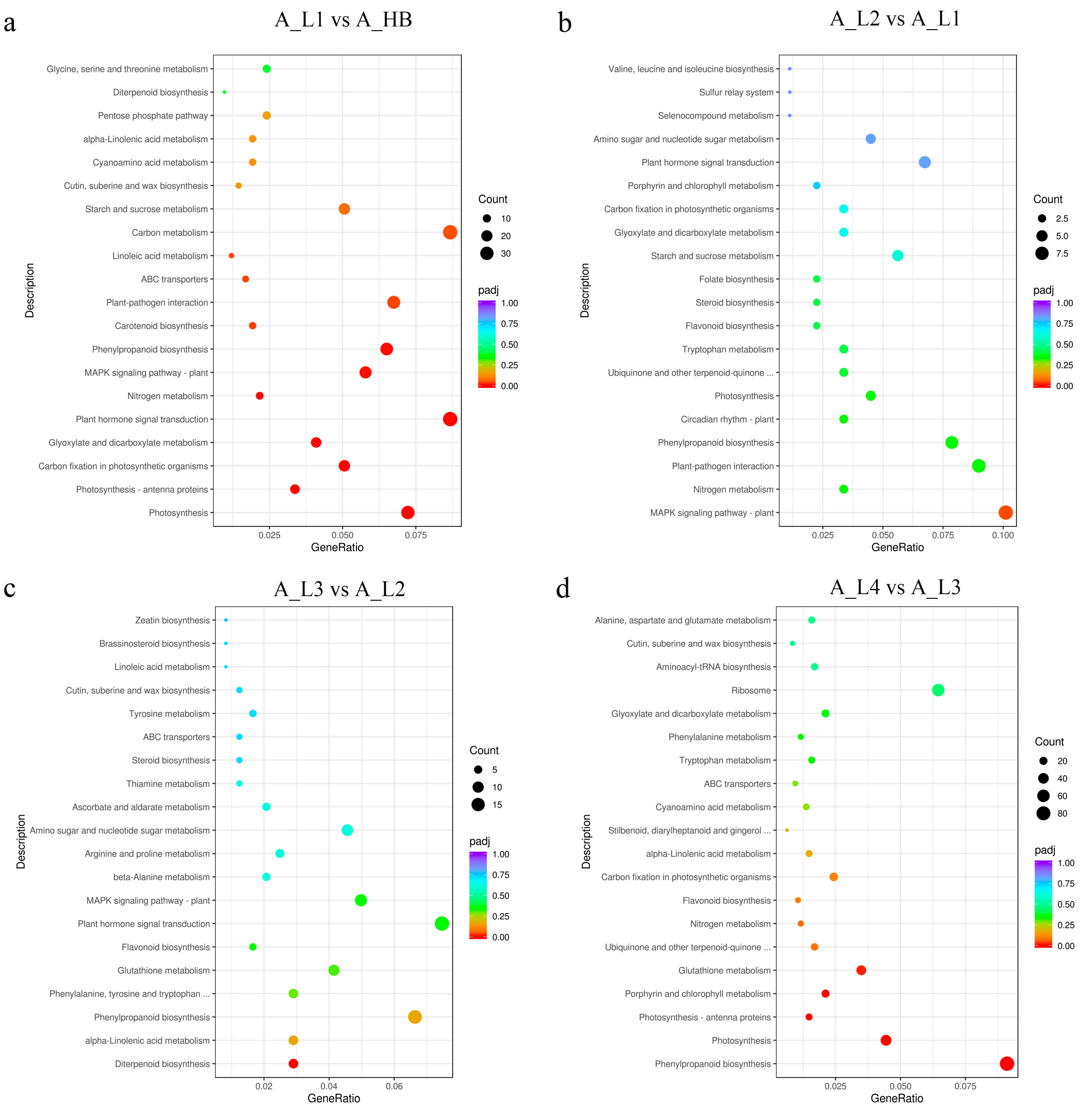

Figure S3. Kyoto Encyclopedia of Genes and Genomes (KEGG) enrichment analysis of the high-tillering genotypes (AKZ-NRGR667). (a) KEGG enrichment of DEGs between A_L1 vs A_HB; (b) GO enrichment of DEGs between A_L2 vs A_L1; (c) GO enrichment of DEGs between A_L3 vs A_L2; (d) GO enrichment of DEGs between A_L4 vs A_L3.

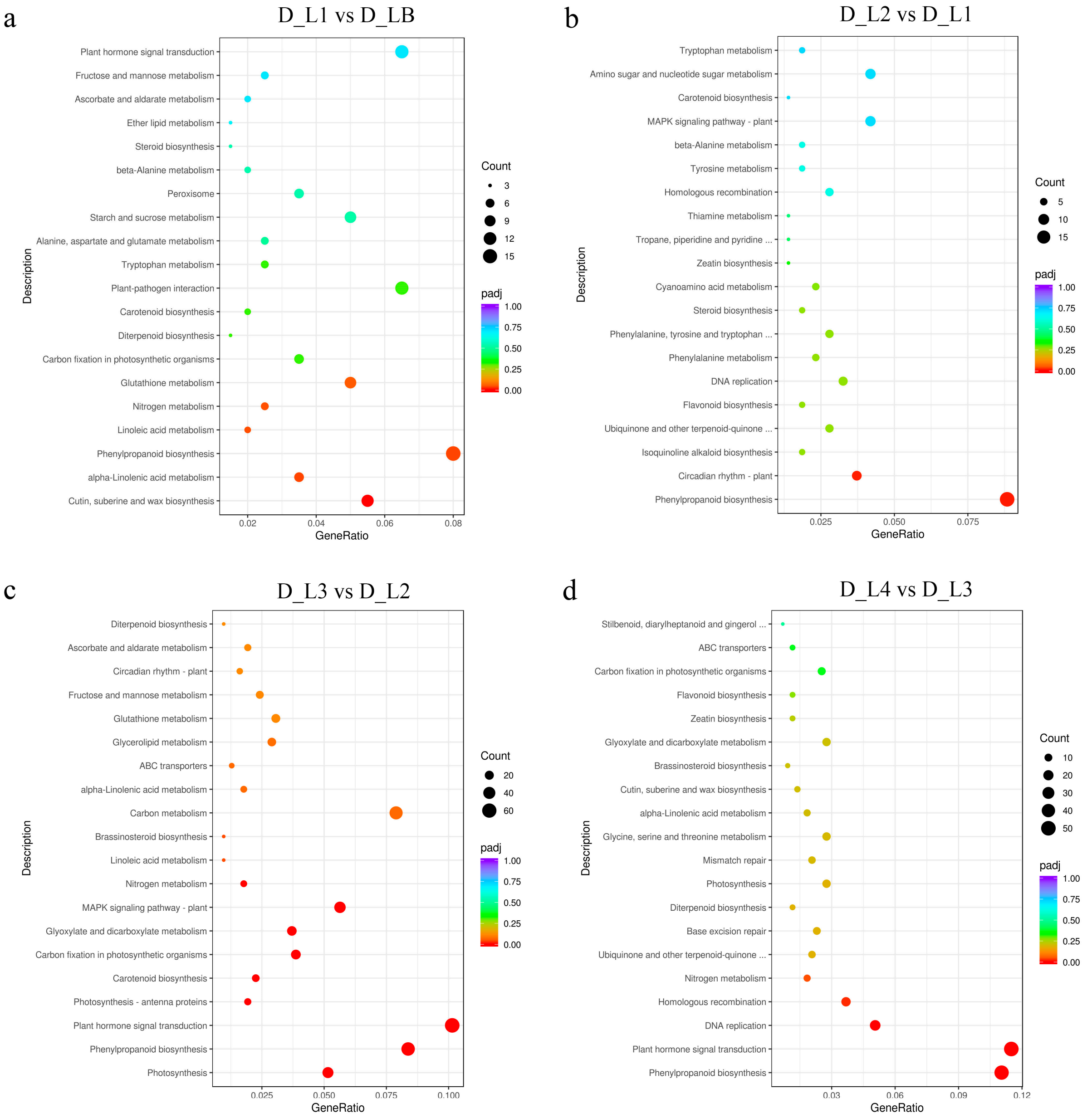

Figure S4. Kyoto Encyclopedia of Genes and Genomes (KEGG) enrichment analysis of the low-tillering genotypes (D20170203). (a) KEGG enrichment of DEGs between D_L1 vs D_HB; (b) GO enrichment of DEGs between D_L2 vs D_L1; (c) GO enrichment of DEGs between D_L3 vs D_L2; (d) GO enrichment of DEGs between D_L4 vs D_L3.

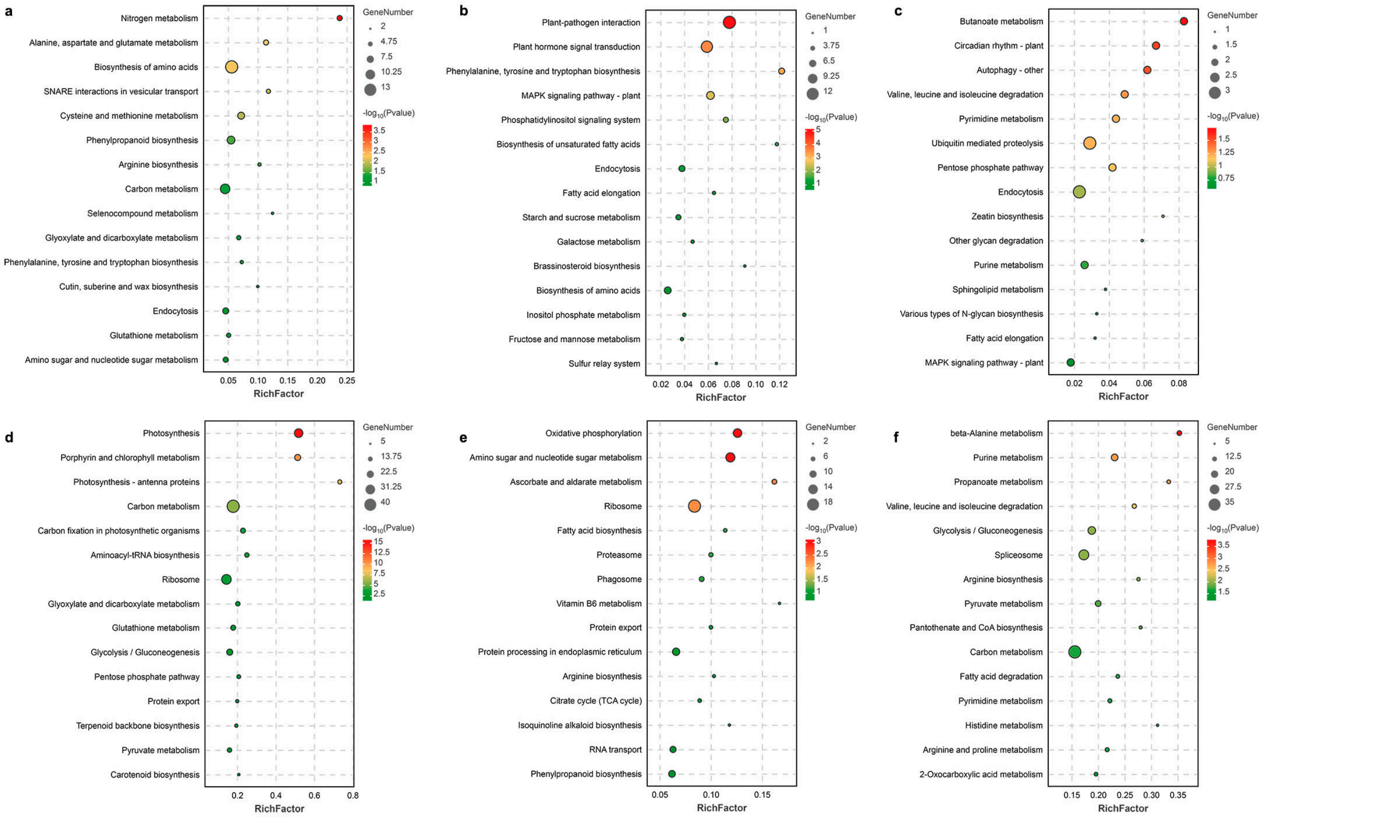

Figure S5. Kyoto Encyclopedia of Genes and Genomes (KEGG) enrichment in different modules. (a) KEGG enrichment of DEGs in pink module; (b) KEGG enrichment of DEGs in magenta module; (c) KEGG enrichment of DEGs in tan module; (d) KEGG enrichment of DEGs in blue module; (e) KEGG enrichment of DEGs in green modules; (f) KEGG enrichment of DEGs in turquoise modules.

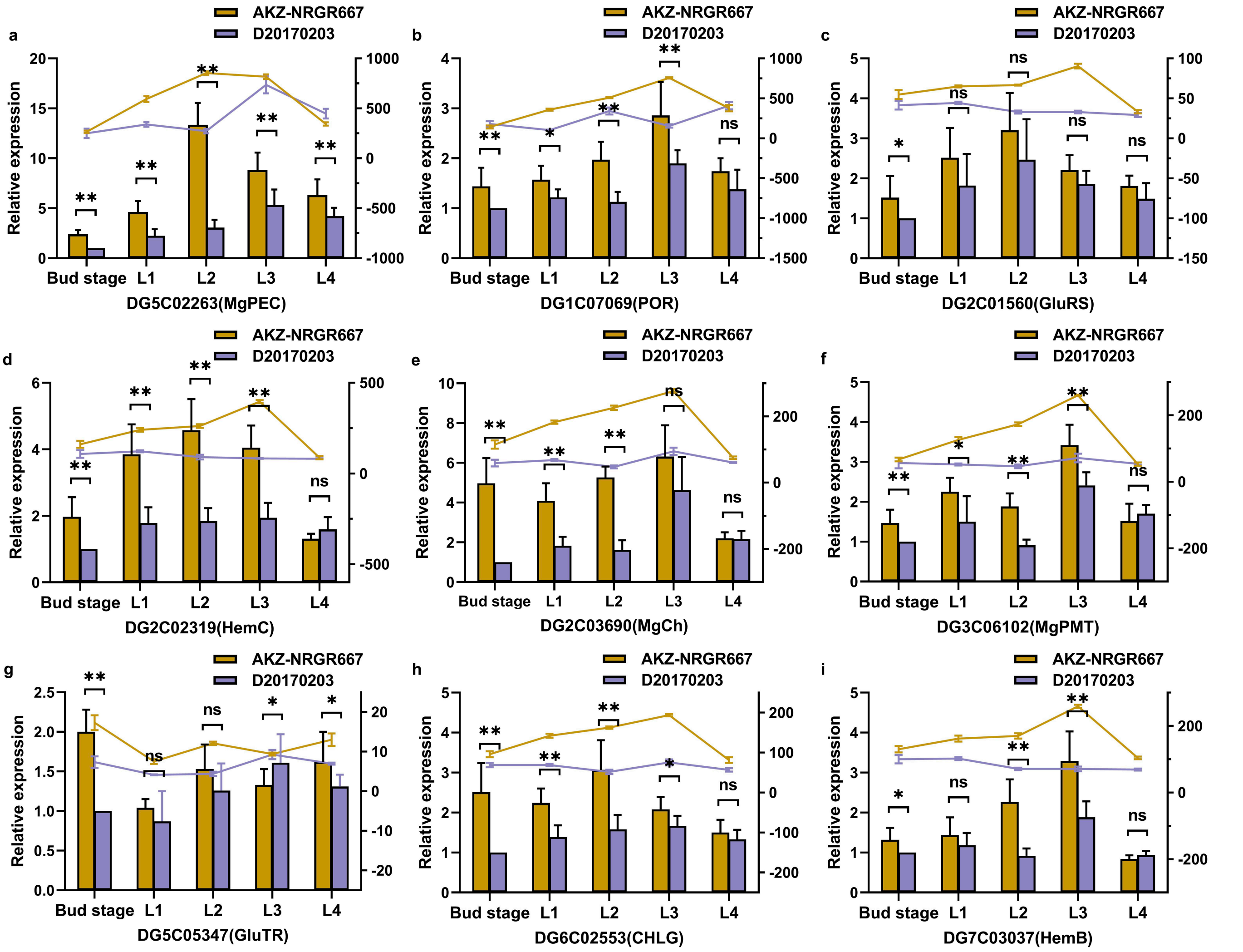

Figure S6. The qRT-PCR expression analysis of nine genes of the chlorophyll biosynthesis pathway at five developmental stages of AKZ-NRGR667 and D20170203. (**a**) MgPEC, magnesium-protoporphyrin IX monomethyl ester cyclase; (**b**) POR, protochlorophyllide reductase; (**c**) GluRS, glutamate-tRNA ligase; (**d**) HemC, porphobilinogen deaminase; (**e**) MgCh, magnesium chelatase subunit H; (**f**) MgPMT, magnesium protoporphyrin IX methyltransferase; (**g**) GluTR, glutamyl-tRNA reductase (**h**) CHLG, Chlorophyll synthase ; (**i**) HemB, ALA dehydratase. The brown column denotes AKZ-NRGR667, and the purple column denotes D20170203. The brown line denotes the RNA-seq data of AKZ-NRGR667, and purple denotes that of D20170203. "ns" indicates no significant difference, "*" indicates the statistical significance at p-value < 0.05, "**" indicates the statistical significance at *p*-value < 0.01.
